# Immediate Breast Reconstruction with Fat-Graft-Augmented ICAP Flaps

**DOI:** 10.3390/life15071017

**Published:** 2025-06-26

**Authors:** Francesco Klinger, Mattia Federico Cavallero, Andrea Vittorio Emanuele Lisa, Fernando Rosatti, Barbara Catania, Marco Klinger, Riccardo Di Giuli, Simone Furlan, Roberta Comunian, Stefano Vaccari, Valeriano Vinci

**Affiliations:** 1 Department of Health Sciences, Ospedale San Paolo, University of Milan, via Antonio di Rudinì 8, 20142 Milan, Italy; 2 Department of Medical Biotechnology and Translational Medicine BIOMETRA, Plastic Reconstructive and Aesthetic Plastic Surgery School, Università degli Studi Di Milano, via Festa del Perdono 7, 20122 Milan, Italy; 3 IRCCS Humanitas Research Hospital, via Manzoni 56, Rozzano, 20089 Milan, Italy; 4 Department of Plastic and Reconstructive Surgery, European Institute of Oncology, IRCCS, 20139 Milan, Italy; 5 Department of Surgical Sciences, University of Rome “Tor Vergata”, Cracovia n. 50, 00133 Rome, Italy; 6 Department of Biomedical Sciences, Humanitas University, via Rita Levi Montalcini 4, Pieve Emanuele, 20090 Milan, Italy

**Keywords:** breast surgery, breast conservative surgery, immediate breast reconstruction, autologous breast reconstruction, ICAP flap, fat-grafting, fat-graft-augmented ICAP flap

## Abstract

**Background:** Intercostal artery perforator (ICAP) flaps are a reliable option for volume replacement in breast-conserving surgery (BCS), particularly for lower pole defects. However, limited flap volume may reduce their applicability in selected patients. Autologous fat grafting has been proposed to enhance both volume and aesthetic outcomes. **Methods:** This retrospective study evaluated 10 patients undergoing BCS with immediate reconstruction using fat-graft-augmented ICAP flaps. Nine anterior ICAP (AICAP) flaps and one lateral ICAP (LICAP) flap were employed. The outcomes included flap viability, complications, and aesthetic results over a 6-month follow-up. **Results:** Partial flap resorption occurred in 2 patients (20%), both of whom were active smokers. No cases of skin necrosis were observed. Fat grafting volumes ranged from 20 to 60 cc. Aesthetic outcomes were satisfactory, with good restoration of the breast contour and stable integration of the flap and grafted fat. **Conclusions:** The combined use of ICAP flaps and autologous fat grafting is a feasible and effective technique for immediate reconstruction after BCS. It allows volume enhancement, maintains natural contour, and shows low complication rates in properly selected patients. Smoking remains a significant risk factor.

## 1. Introduction

The integration of oncoplastic techniques into breast-conserving surgery (BCS) has significantly improved aesthetic outcomes in women undergoing partial mastectomy.

In this context, intercostal artery perforator (ICAP) flaps have emerged as a versatile option not only for breast reconstruction but also for breast ptosis correction in post-bariatric patients [1], as well as for reconstructing distant defects of the thorax, sacrum, and axillary region and as free flaps [2].

ICAP flaps are based on perforators from the intercostal vessels, which form an arterial arcade between the aorta and the internal mammary vessels, dividing into vertebral, intercostal, intermuscular, and rectus segments [3].

They are classified into dorsal (DICAP), lateral (LICAP), and anterior (AICAP) types based on the source of the perforators. LICAP flaps are suitable for lateral thoracic defects or can be extended posteriorly for larger reconstructions, while AICAP flaps are ideal for inferomedial breast defects [4].

Microsurgical breast reconstruction can present challenges such as vascular compression by the chest wall [5], impaired skin sensitivity [6], and limited donor-site volume⁷. In this setting, autologous fat grafting serves as an effective adjunct, enhancing outcomes and aesthetic results, as demonstrated in the literature [5,6,7]. Notably, the flap acts as a vascularized scaffold that supports fat graft integration [8].

This study investigates the combined use of ICAP flaps and autologous fat grafting in breast reconstruction.

By examining clinical outcomes and synthesizing the available evidence, we aim to evaluate the feasibility, benefits, and potential limitations of this innovative approach.

## 2. Patients and Methods

This retrospective study evaluated 10 patients (10 breasts) who underwent BCS for tumors localized in the inferior breast quadrants, with immediate volume reconstruction using ICAP flaps. Among the 10 ICAP flaps performed, 9 were AICAP flaps and only 1 was a LICAP flap. In two cases, a skin island was preserved in the flap project to reconstruct the skin resection, and in one case, the ICAP flap reconstruction was performed in conjunction with an inverted T mastopexy; autologous fat grafts were used in all cases.

The procedures were performed between 2024 and 2025 at the Humanitas Research Hospital, Milan, Italy.

Patients eligible for this procedure met the following inclusion criteria: (a) having undergone conservative breast surgery, (b) with tumors localized in the inferior breast quadrants, and (c) identification of a well-vascularized perforator artery with an adequate caliber and flow during preoperative color Doppler ultrasound evaluation. Exclusion criteria included a BMI < 20 kg/m^2^, diabetes, poor scarring history, and heavy smoking habits.

Preoperative pedicle assessment involved using a Doppler probe (iU22 xMATRIX ultrasound system with a Linear 12–5 MHz transducer, Phillips Electronics, Andover, MA, USA) and direct visualization. Although computed tomography angiography can visualize the pedicle, it was deemed unnecessary for preoperative evaluation.

Intraoperative and postoperative flap viability was assessed through ultrasound evaluation and clinical evaluation conducted, respectively, at 12 and 24 h and at 24–48 h, on postoperative days 7 and 14, and at 1, 3, and 6 months post-surgery.

Data collection included demographic characteristics of the study population, complication rates, and photographic documentation.

## 3. Operative Technique

**Figure 1:**
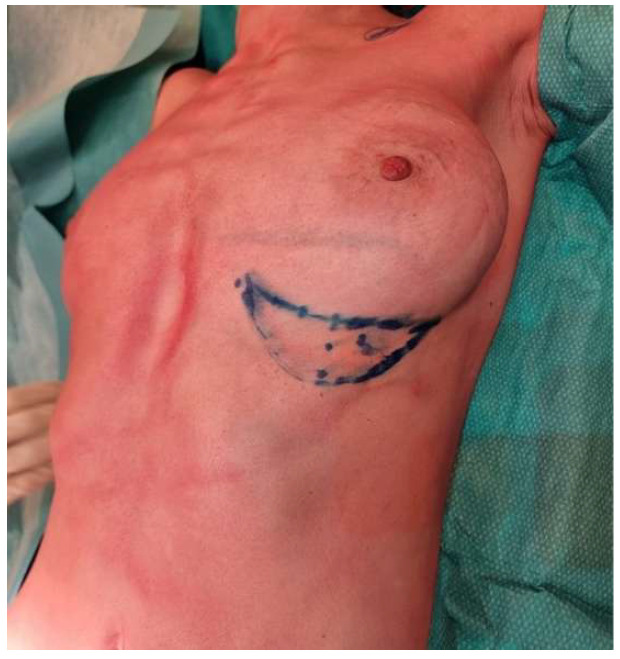
Preoperative marking demonstrating the planned surgical incision, including precise delineation of the flap margins to ensure optimal flap design and accurate tissue mobilization.

The flap is designed along the identified perforator’s axis (Figure 1). In this context, preoperative imaging such as Doppler ultrasound is used to locate the dominant perforators.

Anesthesia is performed with particular care to avoid compressive positioning or infiltration near the perforator vessels, which are preoperatively identified and mapped through preoperative imaging using Doppler ultrasound to preserve their integrity throughout the procedure. A total of 10 mL of levobupivacaine 7.5% is selectively infiltrated to facilitate tissue dissection for flap elevation and to reduce postoperative pain, ensuring both surgical precision and patient comfort.

**Figure 2:**
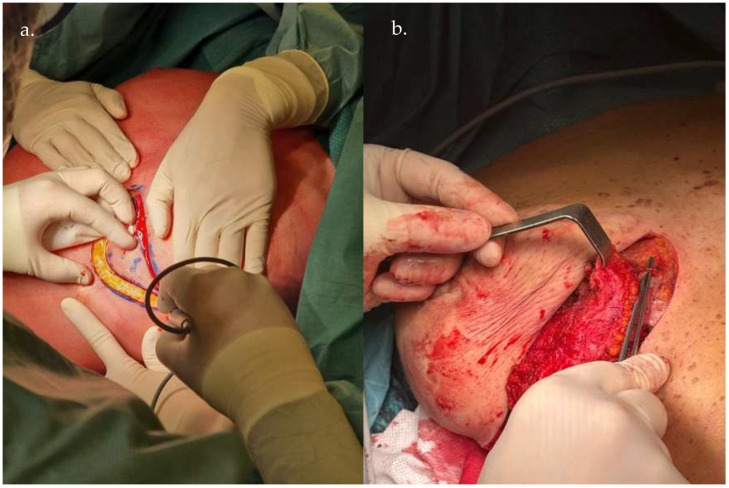
Intraoperative images showing the flap preparation, highlighting the meticulous dissection along the perforator axis (**a**) in order to preserve vascular integrity (**b**).

**Figure 3:**
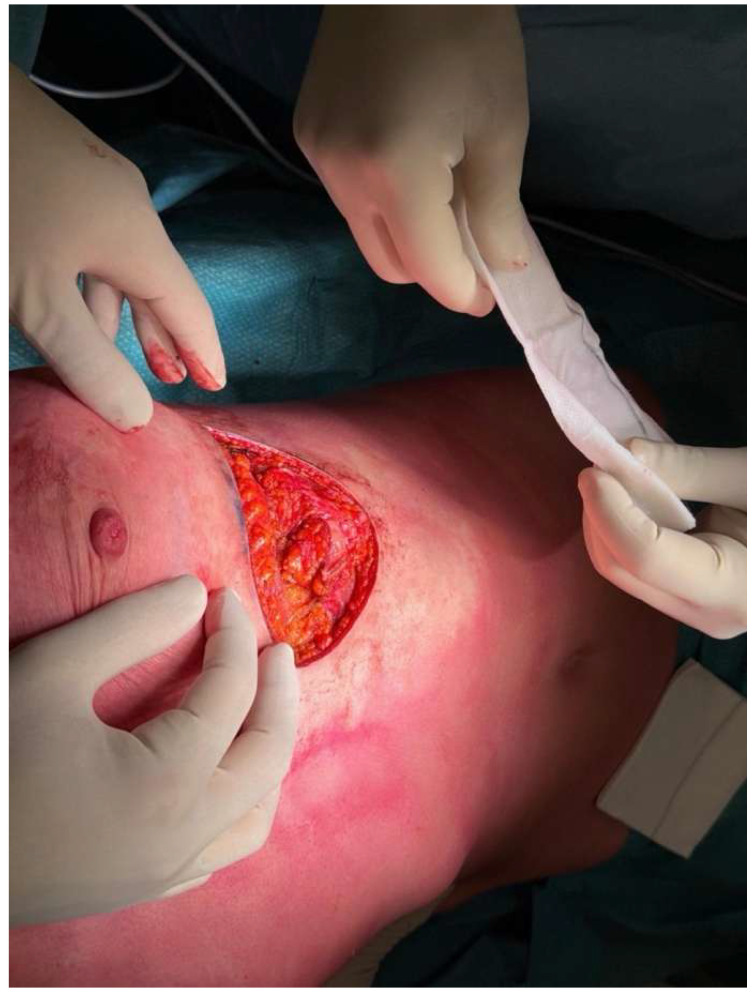
The flap is elevated while maintaining adequate pedicle length for optimal rotation and inset flexibility.

The LICAP or AICAP variant is selected based on the defect’s location. The perforator dissection is meticulously performed to preserve vascularity and allow sufficient pedicle length for rotation and inset flexibility (Figure 2 and Figure 3).

In cases where skin island reconstruction is necessary, the flap may be left non-de-epithelialized to maintain a skin paddle for coverage. This approach allows for the preservation of a viable skin component, essential when reconstructing larger skin defects.

Based on aesthetic considerations, the flap axis is designed to align with the inframammary sulcus, ensuring that the resulting scar is positioned discreetly under the brassiere strap.

Adipose tissue is harvested from the donor site in volumes ranging from 20 to 60 cc using a blunt cannula under low-pressure vacuum in order to preserve cell integrity. In our case series, the donor site was consistently located at the hips.

**Figure 4:**
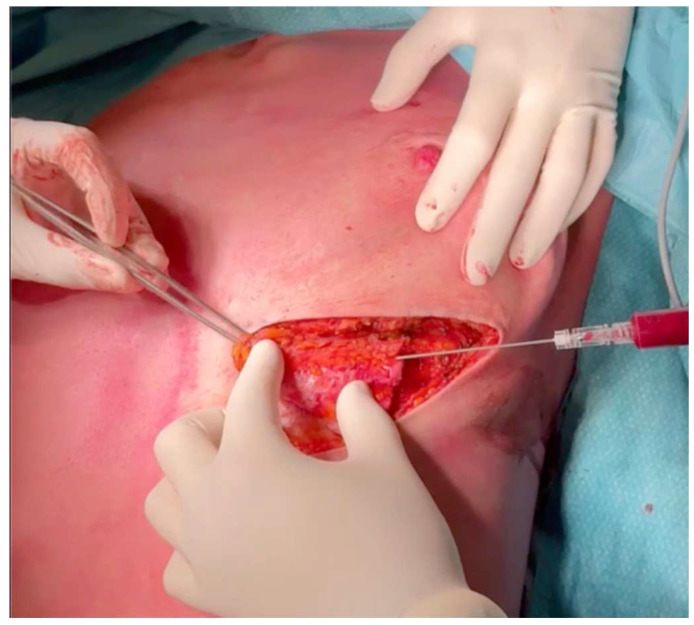
Autologous fat grafting flap augmentation, applying a blunt cannula for precise fat deposition, aiming to enhance volume and contour while minimizing tissue trauma.

The harvested fat is processed using the Coleman technique [9]. Fat is injected with a blunt cannula to minimize trauma (Figure 4), with fat injection in small aliquots while withdrawing the cannula to create microdroplets and complementing the ICAP flap structure.

To optimize breast reshaping, the ICAP flap can be folded or “rolled” onto itself during the inset. This technique increases the projection and fullness of the reconstructed breast mound.

The inset of the flap is stabilized using Vicryl 2/0 sutures to secure the flap to the pectoralis major fascia, maintaining its intended position, while careful attention must be paid to avoid kinking or compression of the vascular pedicle to maintain adequate perfusion. Following stabilization, Vicryl 2/0 sutures are placed in layers. The final step involves a continuous intradermal closure of the skin and a breast elastic taping with a moderate grade of compression.

Patients are closely monitored during their hospital stay to assess flap viability and detect any early complications. After discharge, weekly outpatient visits are conducted during the first month. Follow-up appointments are then gradually spaced out to ensure long-term outcomes at 1, 3, and 6 months post-surgery.

## 4. Results

All the patients in the study underwent ICAP flap fat-augmented volume restoration. In one case, a concomitant inverted T mastopexy was performed due to grade III ptosis, according to the Regnault classification [10]. The mean patient age was 55 (range 40–76) years, and the mean BMI was 24.7 (21.6–29.8) Kg/m^2^. The mean size of the flap was approximately 13.4 × 9.6 cm and the average flap thickness was 2.1 (range 1.7–2.4 cm), as visible in Table 1. In all cases where skin reconstruction was performed using a non-de-epithelialized flap, no signs of skin necrosis were observed postoperatively.

Among the 10 patients included in the study, 2 were active smokers and 1 was a former smoker.

All flaps were clinically well-vascularized after complete elevation and were successfully transferred, although partial flap loss was seen in 20% of the cases.

The brassiere cup size remained unchanged in all patients.

## 5. Clinical Examples

**Figure 5:**
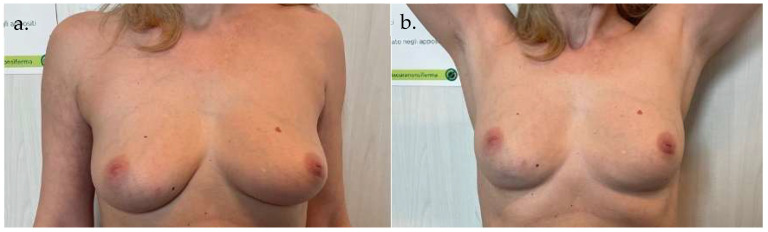
Preoperative images of Case 1. (**a**) Frontal view of the breast region. (**b**) Frontal view including upper arms for assessment of body contour and symmetry.

**Figure 6:**
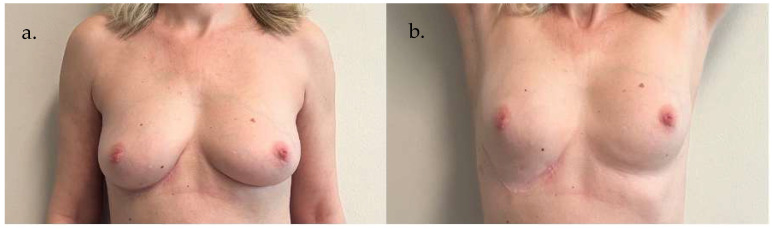
Postoperative images of Case 1, taken 60 days after surgery. (**a**) Frontal view showing breast reconstruction outcome. (**b**) Frontal view with upper arms to illustrate overall aesthetic integration.

**Case 1 (Figure 5 and Figure 6):** A 55-year-old woman underwent an infero-external and partial infero-internal right breast quadrantectomy followed by plastic reconstruction using an AICAP flap. The flap measured 12 cm × 6 cm and was approximately 2 cm thick. The donor area was directly closed, and the flap was de-epithelialized before being positioned to cover the breast mound. Photographs were taken 60 days postoperatively.

**Figure 7:**
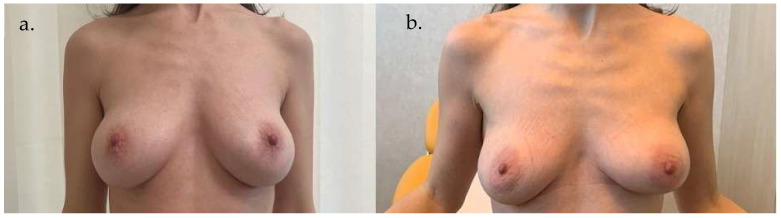
Preoperative (**a**) and postoperative (**b**) images, taken 120 days after surgery, of Case 2. Frontal view illustrating the tumor location and breast morphology prior to quadrantectomy and reconstruction (**a**). Frontal view demonstrating flap integration and left breast contour restoration (**b**).

**Case 2 (Figure 7):** A 38-year-old woman underwent an infero-external left breast quadrantectomy breast cancer, followed by immediate volume replacement using a fat-augmented AICAP flap. The flap measured 15 cm × 12 cm and was approximately 2.2 cm thick. After careful elevation and de-epithelialization, the flap was positioned to restore the breast contour, effectively covering the surgical defect. The donor area was directly closed without tension. Postoperative recovery was uneventful, and photographs were taken 120 days after surgery, documenting a satisfactory aesthetic outcome and stable flap integration.

## 6. Discussion

Nowadays, numerous oncoplastic techniques have been developed to excise small to medium-sized tumors while ensuring an adequate safety margin and achieving favorable outcomes [11,12,13]. Although various local techniques are available, selecting the appropriate approach requires careful consideration of multiple factors.

The use of the intercostal neurovascular pedicle to supply skin flaps is a well-established concept in flap surgery, first proposed by Esser et al. [14] in 1931. In 1974, Dibbell et al. [15] reported a clinical case involving a young paraplegic patient with a sacral bed sore. In this case, an intercostal flap incorporating the anterior cutaneous nerve was utilized to provide sensation to the sacral region. Subsequently, Daniel et al. [16] included the lateral cutaneous branch of the intercostal nerve in musculocutaneous flaps. These flaps were harvested as musculocutaneous constructs with random extensions beyond the thoracic cage, often requiring delay procedures to improve their reliability. The extensive anatomical studies conducted by Kerrigan and Daniel [2,17] contributed significantly to refining the clinical indications and surgical techniques for these flaps.

As described by Hamdi et al. [18], these flaps can be based on an intercostal perforator arising from either the costal or muscular segments of the intercostal vessels, enabling the surgeon to construct a reliable skin paddle for chest wall and breast reconstruction. According to these authors [18], these dominant perforators were located on average 3.5 cm from the anterior border of the latissimus dorsi muscle and in the fourth to eighth intercostal spaces, with a higher concentration in the sixth and seventh intercostal spaces. When planned and performed effectively, this anatomical characteristic permits adequate flap vascularization and minimizes vascular pedicle complications [19].

In the present study, ICAP flaps were used to repair lateral and central-inferior skin and breast tissue resected during conservative breast surgery (CBS) in the context of patients with a small to medium breast volume [20]. We observed that using tissue adjacent to the defect provided matching texture and color to the original breast. In addition, it is important to design the skin island flap according to the tumor location to avoid pedicle tension. The utility of the ICAP flap for volume replacement in breast reconstruction has been well documented in the literature [3,21,22,23,24,25,26]. However, their volume may be insufficient in larger defects, requiring careful patient selection or combination with other flaps [27].

In this context, autologous fat grafting is a safe and effective tool for secondary breast reconstruction, enhancing contour, volume, and overall breast shape and symmetry [28]. This technique is particularly beneficial in breast reshaping, as evaluated by Klinger et al. for composite breast augmentation [29] and for secondary contour deformities, providing a simple and low-morbidity solution [30]. Moreover, autologous fat grafting has been shown to be a relatively safe procedure for refining reconstructed breasts in postmastectomy patients, with low complication rates and no observed cases of locoregional cancer recurrence [31,32]. Additionally, it has demonstrated beneficial effects in alleviating postmastectomy pain syndrome following breast-conserving surgery, likely due to its regenerative and anti-inflammatory properties [33].

To overcome the volume limitations of the ICAP flap in larger defects, the fat-graft-augmented ICAP flap has been introduced, combining perforator flap reliability with autologous fat grafting to enhance volume and contour restoration.

The study by Laporta et al. [7] evaluated the effectiveness of breast reconstruction using DIEP (deep inferior epigastric perforator) flaps augmented with autologous fat grafting in patients with insufficient donor-site volume. The findings indicate that this technique is both feasible and safe, providing satisfactory aesthetic outcomes without increasing the number of secondary procedures or extending the overall treatment duration compared to DIEP flap reconstruction without fat grafting [4].

In this context, as shown in Table 2, the overall complication rate was 20%, with no cases of tissue necrosis. The only complication observed was partial flap resorption, which occurred in two cases, both belonging to the active smoker group. Demiri EC et al. [34] reported an overall complication rate of 38.8%, with only one case of fat necrosis observed in the fat-augmented latissimus dorsi group. The lower flap resorption rate observed in our study can be attributed to the smaller flap volume and the consequently reduced amount of fat augmentation required to achieve the expected surgical result. The absence of tissue necrosis observed in our cohort further supports the stability and reliability of the flap’s vascular supply, a concept corroborated by previous studies highlighting the robust perfusion and low complication rates associated with well vascularized perforator flaps [35].

Obviously, care must be taken in high-risk patients, such as smokers.

As visible in Table 2, the complication rate and the total of cases of partial flap loss were higher in the smoker group, confirming the impact of smoking on postoperative outcomes in breast reconstruction [36].

The limitations of this study include the relatively small number of patients, which may affect the generalizability of the findings. Additionally, the retrospective nature of the study and the lack of an extended follow-up period limit the ability to comprehensively evaluate long-term outcomes and potential late complications. Furthermore, being a single-center study, the findings may reflect institution-specific factors, potentially limiting broader applicability.

## 7. Conclusions

By combining ICAP flaps with autologous fat grafting, surgeons can achieve natural and harmonious breast reconstructions, leveraging the advantages of both techniques to optimize aesthetic and functional outcomes. The overall complication rate in this study was low, supporting the safety profile of the procedure. Notably, no cases of tissue necrosis were observed, underscoring the reliability of the vascular pedicle and its capacity to sustain adequate perfusion, including support for the survival of the transferred autologous fat. Furthermore, complications were observed exclusively in the smoker group, highlighting a statistically significant correlation between smoking and adverse postoperative outcomes. These findings reinforce the role of ICAP flaps augmented with fat grafting as a reliable and safe option for immediate reconstruction following CBS and emphasize the importance of patient selection and preoperative counseling regarding modifiable risk factors such as smoking.

## Figures and Tables

**Table 1:** Demographic and clinical characteristics of the study population. This table summarizes the baseline characteristics of all patients included in the study. Data include patient age (years), type of surgical intervention (AICAP or LICAP flap), body mass index (BMI, kg/m^2^), and smoking status (active smoker, former smoker, non-smoker). These parameters provide an overview of the clinical context and potential risk factors influencing surgical outcomes.

AGE (y.o.)	AICAP or LICAP	BMI (kg/m^2^)	SMOKING
65	AICAP	29.76	no
65	AICAP	25.64	yes
45	AICAP	18.22	no
39	AICAP	20.81	no
47	AICAP	19.61	yes
76	AICAP	26.84	no
57	AICAP	21.56	no
66	LICAP	24.03	no
40	AICAP	23.05	no
49	AICAP	23.11	no

**Table 2:** Comparison of complication rates between smokers and non-smokers. This table presents the distribution of postoperative complications observed in the study cohort, stratified by smoking status. Data include the total number of patients in each group (active smokers vs. non-smokers/former smokers), the incidence of partial flap resorption, fat necrosis, and other complications. The table highlights the higher rate of partial flap resorption in the smoker group, supporting the negative impact of smoking on microvascular perfusion and tissue healing.

	SMOKERS (Total No. 2)	NON-SMOKERS (Total No. 8)	Total (No. 10)
PARTIAL FLAP LOSS (%)	100	0	20
TISSUE NECROSIS (%)	0	0	0

## Data Availability

The original contributions presented in this study are included in the article. Further inquiries can be directed to the corresponding author.
